# Supplementary material for: Femoral Anteversion Measured by the Surgical Transepicondylar Axis Is Correlated with the Tibial Tubercle–Roman Arch Distance in Patients with Lateral Patellar Dislocation
Source: Medicina (Kaunas). 2023 Feb 16;59(2):382. doi: 10.3390/medicina59020382 (PMC9959396; doi:10.3390/medicina59020382)
Supplement: Supplementary file 1 [file medicina-59-00382-s001.zip › Figure S1 knee joint rotation.pdf]

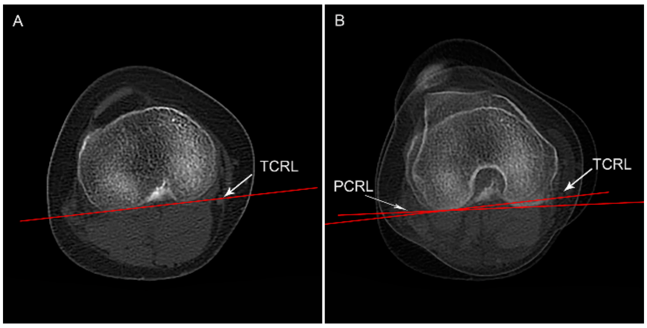

Figure S1. Knee joint rotation. (A) The most proximal axial slice with intact bilateral tibial condyles, showing the line tangent to the condyles (TCRL). (B) The posterior femoral condylar reference line (PCRL) is shown on the slice showing intact femoral condyles and Roman arch. Superimpose these two images, the angle between TCRL and PCRL is defined as knee joint rotation.
