# Supplementary material for: Femoral Anteversion Measured by the Surgical Transepicondylar Axis Is Correlated with the Tibial Tubercle–Roman Arch Distance in Patients with Lateral Patellar Dislocation
Source: Medicina (Kaunas). 2023 Feb 16;59(2):382. doi: 10.3390/medicina59020382 (PMC9959396; doi:10.3390/medicina59020382)
Supplement: Supplementary file 1 [file medicina-59-00382-s001.zip › Figure S2 patellar height.pdf]

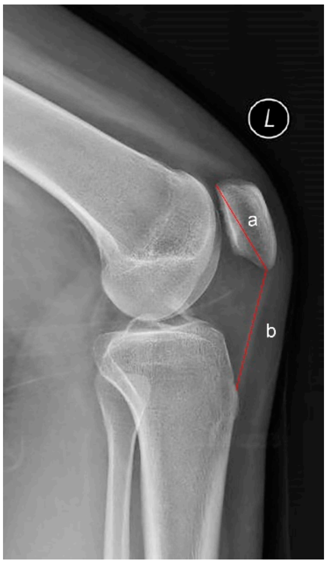

Figure S2. Insall-Salvati index (ISI) for evaluating patellar height. It is defined as the ratio of the patellar tendon (b) to maximum length measured from the distal pole to the proximal pole of the patella (a).
